# Supplementary material for: Hericium erinaceus Extract Exerts Beneficial Effects on Gut–Neuroinflammaging–Cognitive Axis in Elderly Mice
Source: Biology (Basel). 2023 Dec 28;13(1):18. doi: 10.3390/biology13010018 (PMC10813749; doi:10.3390/biology13010018)
Supplement: Supplementary file 1 [file biology-13-00018-s001.zip › Tables S1 and S2.pdf]

Table 1. Primary antibodies employed for microscopy experimental procedures.

	Antigen	Immunogen	Manufacturer, Species, Mono-Polyclonal, Catalogue or Lot No., RRID	Diluted Used
**Primary antibodies**	Anti-CD45	Purified antibody raised against a carrier-protein conjugated synthetic peptide encompassing a sequence within the C-terminus region of human CD45	GeneTex, Inc. (North America, USA), Rabbit polyclonal IgG, Cat# Cat# GTX116018, RRID: AB_10621655470	1:200
	Anti-Interleukin-6 (M-19)	Purified antibody raised against a peptide mapping at the C-terminus of murine IL6	Santa Cruz Biotechnology (Santa Cruz, CA, USA), Goat polyclonal IgG, Cat# sc-1265, RRID: AB_2127470	1:200
	Anti-Glial fibrillary acidic protein (C-19)	Purified antibody raised against a peptide mapping at the C-terminus of GFAP of human origin	Santa Cruz Biotechnology (Santa Cruz, CA, USA), Rabbit polyclonal IgG, Cat# sc-6170, RRID: AB_641021	1:100
	Anti-p62/SQSTM1	Recombinant full-length protein corresponding to Human SQSTM1/p62 aa 1-440	Abcam (Cambridge, United States), Mouse monoclonal, Cat# ab56416, RRID: AB_945626	1:100
	Anti-Histone H2A.X (6L16)	Purified antibody raised against KLH-conjugated linear peptide corresponding to 9 amino acids surrounding phospho-serine 139 of human Histone H2AX	Merck KGaA (Darmstadt, Germany), Rabbit Monoclonal Antibody, Cat# ZRB05636, RRID: AB_309864.	1:100

Table 2. Secondary antibodies employed for microscopy experimental procedures.

	Antigen	Immunogen	Manufacturer, Species, Mono-Polyclonal, Catalogue or Lot No., RRID	Diluted Used
**Secondary antibodies**	Biotinylated goat anti-rabbit IgG	Gamma immunoglobulins	Vector Laboratories (Burlingame, CA, USA), Goat, lot# PK-6101, RRID: AB_2336820	1:200
	Alexa Fluor™ 594 donkey anti-goat IgG (H + L) Highly Cross-Adsorbed Secondary Antibody	Gamma Immunoglobins Heavy and Light chains	Thermo Fisher Scientific (Monza, Italy)	1:200
	Alexa Fluor™ 488 goat anti-rabbit IgG (H + L) Highly Cross-Adsorbed Secondary Antibody	Gamma Immunoglobins Heavy and Light chains	Thermo Fisher Scientific (Monza, Italy)	1:200
