# Supplementary figures and images for: Hericium erinaceus Extract Exerts Beneficial Effects on Gut–Neuroinflammaging–Cognitive Axis in Elderly Mice
Source: Biology (Basel). 2023 Dec 28;13(1):18. doi: 10.3390/biology13010018 (PMC10813749; doi:10.3390/biology13010018)

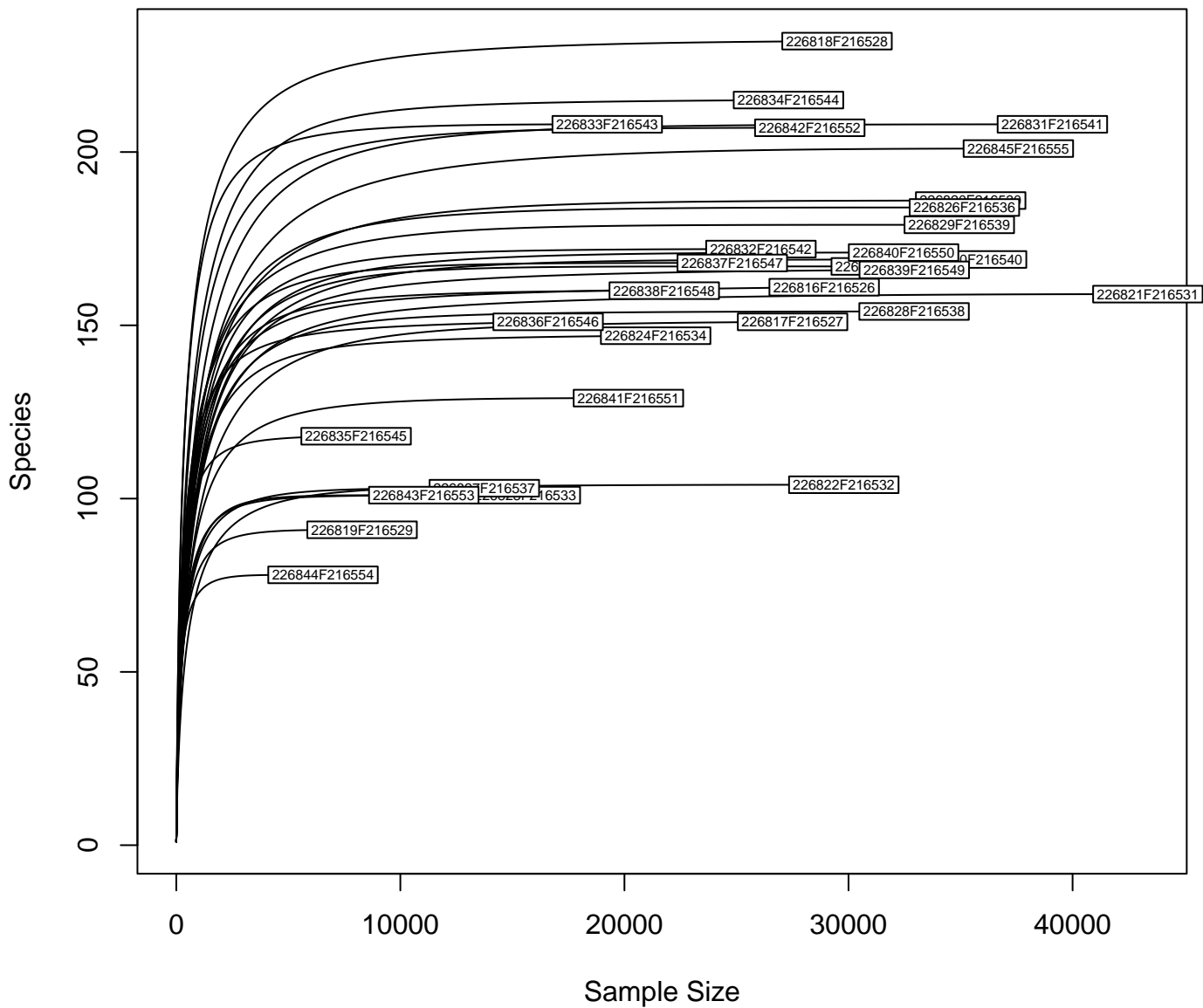

Supplement: Supplementary file 1 [file biology-13-00018-s001.zip › Figure S1.pdf]

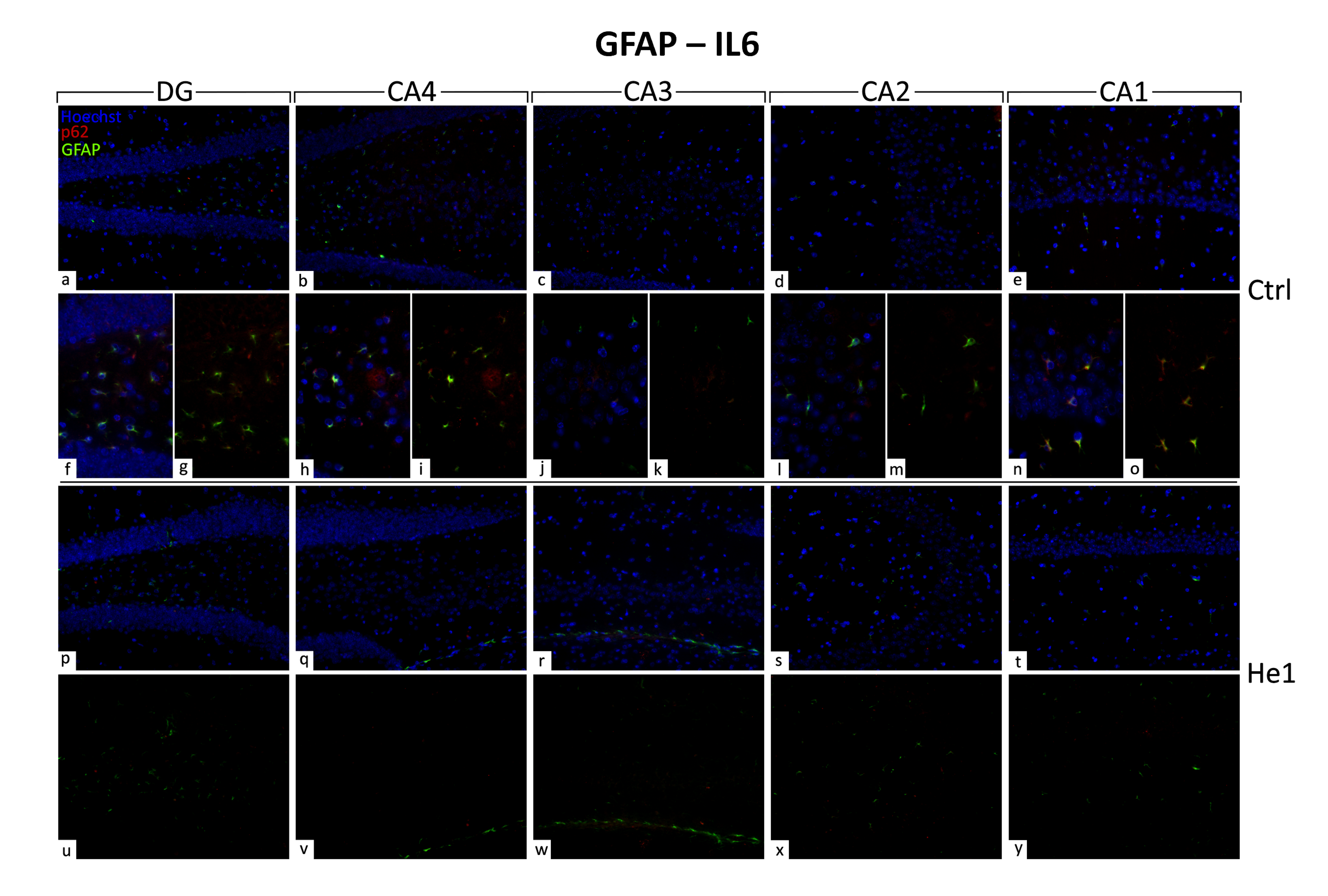

Supplement: Supplementary file 1 [file biology-13-00018-s001.zip › Figure S2.tif]

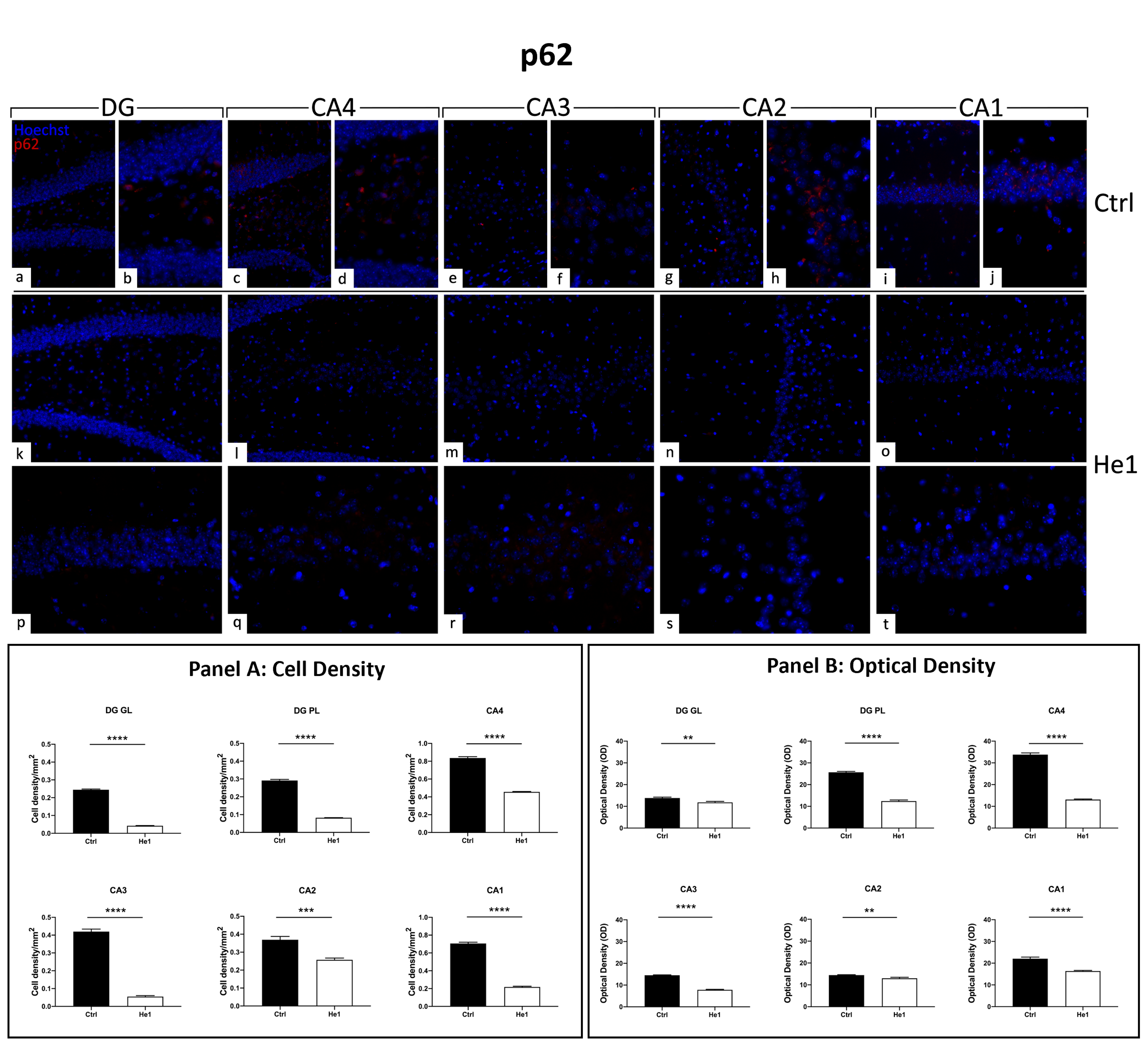

Supplement: Supplementary file 1 [file biology-13-00018-s001.zip › Figure S3.tif]
